# Superficial Angiomyxoma of Axilla: A Case Report

**DOI:** 10.7759/cureus.48472

**Published:** 2023-11-07

**Authors:** Trupti Tonape, Sreedhanya Sv

**Affiliations:** 1 General Surgery, Dr. Dnyandeo Yashwantrao (D.Y. Patil Medical College, Hospital, and Research Centre, Pune, IND

**Keywords:** rare tumors, cutaneous tumors, benign, axillary swelling, superficial angiomyxoma

## Abstract

Superficial angiomyxomas of the skin are rare benign cutaneous tumors of soft tissue composed of myxoid matrix and thin-walled blood vessels. They can be sporadic or develop in conjunction with the Carney complex. These tumors have a predilection for the trunk, lower limbs, head, neck, and genitalia. Herein, we report a case of superficial angiomyxoma of the axilla in a 42-year-old man. The pedunculated polypoidal mass showed a maximum diameter of 4.5 cm and intra- and extra-lesional vascularity on Doppler, and the histopathology report was suggestive of myxoid matrix with scattered bland stellate and spindle cells and long thin-walled branching blood vessels and inflammatory infiltrate consisting of mainly neutrophils. Smooth muscle actin (SMA), S100, and desmin were found to be negative on immunohistochemistry, but CD34 was discovered to be positive. It was possible to make the diagnosis of superficial angiomyxoma using these histological and immunohistochemical characteristics. Wide local excision, being the preferred treatment, was performed, and the patient was followed up for six months with no signs of recurrence.

## Introduction

Superficial angiomyxomas (SA) are benign, infrequent cutaneous tumors that can be either sporadic or develop in conjunction with the Carney complex, particularly if they affect the external ear. They are composed of a distinct myxoid matrix and numerous thin-walled blood vessels. This is more frequently observed in men and has a predilection for the trunk, lower limbs, head, neck, and genitalia, with an incidence of approximately 0.008%-3% [1,2]. Treatment of choice is complete excision, but local recurrence is seen in 3.6-38.1% of cases [1,2]. Differential diagnosis includes fibroma, papilloma, localized neurofibroma, aggressive angiomyxoma, nodular hidradenoma, and cellular angiofibroma. Herein, we report the case of a 42-year-old male with superficial angiomyxoma of the left axilla without carney complex.

## Case presentation

**Figure 1:**
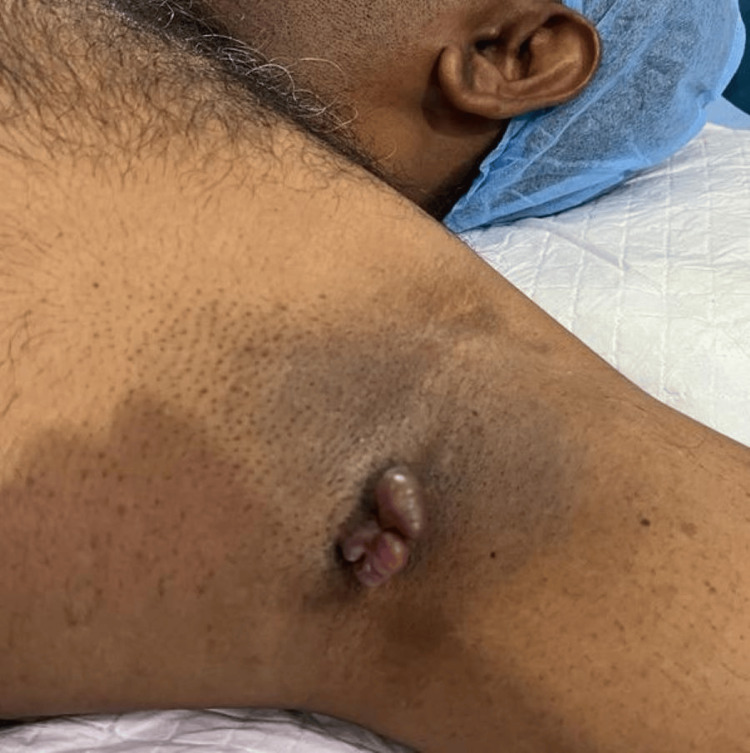
Pre-operative picture of polypoidal swelling in the left axilla

A 42-year-old male reported to the clinic with a history of swelling in the left axilla for one month, gradually progressing in size. The swelling was not associated with pain. There was no history of similar swelling elsewhere in the body and no history of pigmentation of the skin. There was no relevant medical history or family history. Physical examination revealed two flesh-colored, polypoidal masses noted in the left axilla, each measuring roughly 2x3cm in dimension, non-tender with normal skin surrounding it. Figure 1 depicts the polypoidal swelling in the left axilla.

Diagnosis

**Figure 2:**
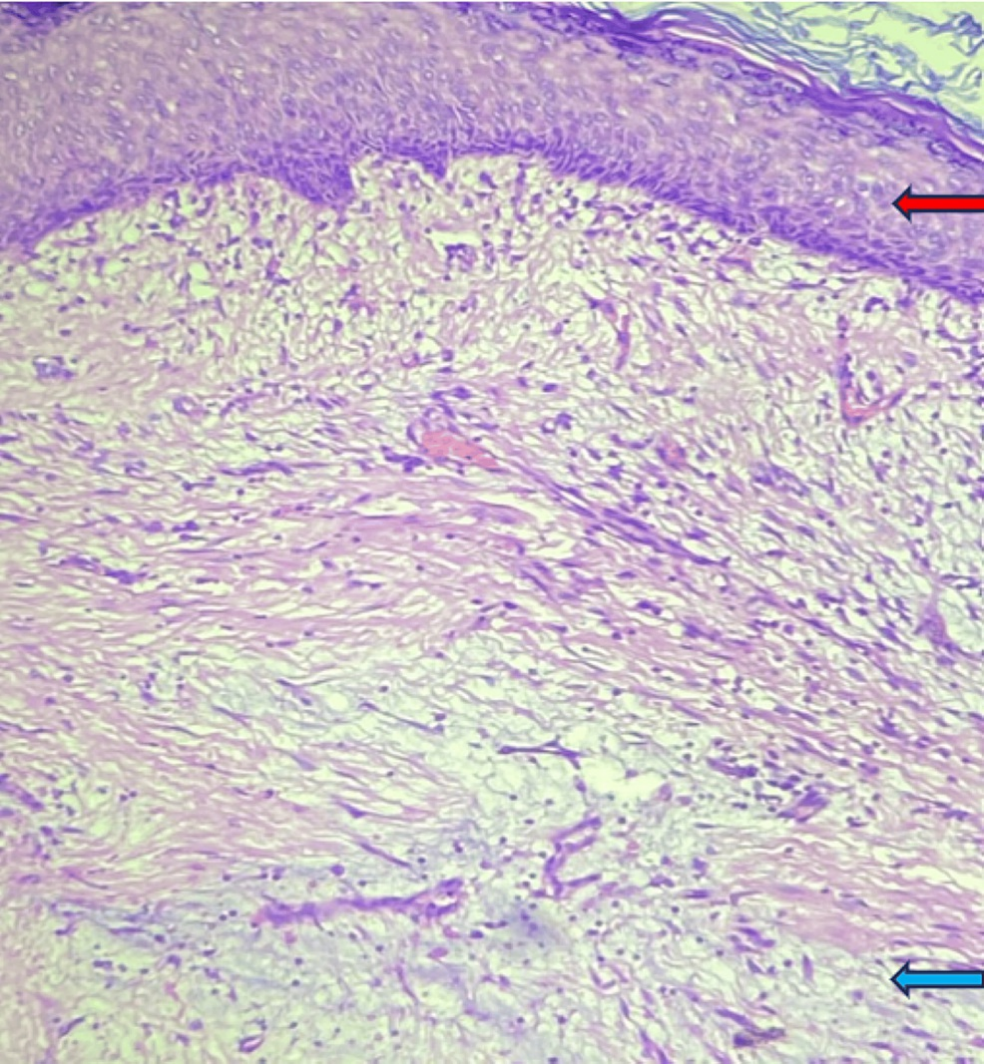
Histopathological image with the red arrow showing keratinizing squamous epithelium and the blue arrow showing a hypocellular myxoid matrix with scattered bland spindle and stellate cells.

Histopathology of the specimen is suggestive of multilobulated polypoidal tumor tissue overlined by flattened keratinizing stratified squamous epithelium and comprised of hypocellular myxoid matrix containing scattered bland spindle and stellate cells and long branching thin-walled blood vessels. Mitotic activity is rare; acellular mucin cysts, entrapped strands of squamous epithelium, and tiny keratinous cysts are evident. Stroma also shows mixed inflammatory infiltrate consisting of neutrophils, lymphocytes, and plasma cells. Immunohistochemistry markers were negative for S100, smooth muscle acin (SMA), and desmin and positive for CD34. In Figure 2, the red arrow shows keratinizing squamous epithelium and the blue arrow shows a hypocellular myxoid matrix with scattered bland spindle and stellate cells. Table 1 depicts the differential diagnosis of superficial angiomyxoma and their positive immunohistochemistry markers.

**Table 1:** Differential diagnosis of superficial angiomyxoma and their positive IHC markers IHC - immunohistochemistry; SF1 - steroidogenic factor 1; SMA - smooth muscle actin; PR - progesterone receptor; ER - estrogen receptor

Differential diagnosis	IHC markers
Fibroma	WT1, SF1, vimentin, CD56, SMA, PR, reticulin positive
Papilloma	-
Localized neurofibroma	S100, SOX10, CD34, collagen type IV strong positivity
Aggressive angiomyxoma	Desmin, SMA, calponin, HMGA2, CDK4 positive, S100 negative
Myxoid neurofibroma	S100, SOX10, CD34, collagen type IV strong positivity
Cutaneous localized mucinosis	-
Nodular hidradenoma	AE1/AE3, SMA partially (stains not particularly helpful)
Cellular angiofibroma	ER, PR, vimentin, SMA, CD34 positive

Treatment

**Figure 3:**
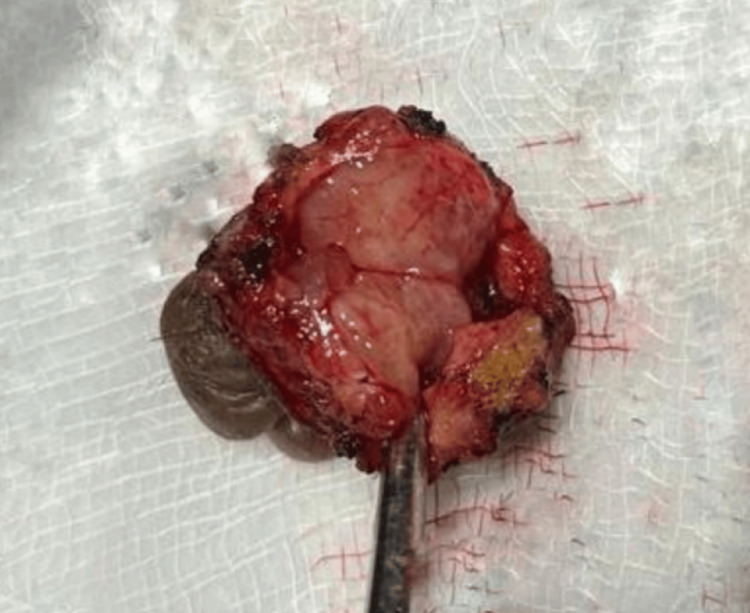
Cut section of the excised axillary mass showing a multilobulated appearance

The preferred treatment is wide local excision of the lesion, which the patient underwent without any complications. An elliptical incision was taken around the swelling in the axilla, with a margin of 5mm, and deepened in layers to excise the swelling. Care was taken to avoid too much dissection to avoid seroma formation. After achieving hemostasis, closure was done in two layers. The postoperative course was uneventful, and the patient showed no signs or symptoms of recurrence after six months at the time of writing this report. Figure 3 shows a cut section of the excised axillary mass showing a multilobulated appearance.

## Discussion

The patient presented with complaints of swelling in the left axilla of one month duration, for which the derived differentials were fibroma, papilloma, and localized neurofibroma. Generally, superficial angiomyxomas invade into the subcutaneous tissue, and an increase in this depth is directly related to an increased risk of recurrence and aggressiveness. The tendency of lesions to recur also increases with the presence of epithelial components, with a recurrence rate of 68% compared to 13% in lesions without epithelial components [2]. Reported clinical differentials of similar swellings over other areas of the body include superficial angiomyxoma, aggressive angiomyxoma, and nodular hidradenoma. The pathological differential diagnosis includes aggressive angiomyxoma, myxoid neurofibroma, cutaneous focal mucinosis, and cellular angiofibroma. In the literature, superficial angiomyxomas have a predilection for the head, neck, trunk, and genitalia [3], whereas in our case, there is an unusual presentation of superficial angiomyxoma in the axilla. Immunohistochemically, CD34 was positive, and desmin, SMA, and S100 were found to be negative, hence distinguishing it from the other differential diagnosis. Treatment of choice is wide local excision of the tumor, but studies show a local recurrence rate of 20% to 30% [4]. Recurrence depends upon various factors such as epithelial components, depth of invasion, and adequacy of excision of the lesion [1-2, 4-5]. The average recurrence time of superficial angiomyxoma is 18 months [2]. For aggressive angiomyxoma, there is an increased recurrence rate of 36% to 72%, and rare metastasis has also been noted [5-6]. Recent advances in the treatment of aggressive angiomyxoma include the use of gonadotropin-releasing hormone agonist, anti-estrogens, and aromatase inhibitor, which seems active in advanced aggressive angiomyxoma but warrants larger studies [7]. Superficial angiomyxomas have an overall good prognosis as these remain superficial without affecting the deeper structures, and the patient needs to be under close follow-up as local recurrence is common. Wide local excision was performed on this patient, and the patient is under close follow-up and remains relapse-free even after six months at the time of writing this article.

## Conclusions

In summary, a rare case of superficial angiomyxoma with an unusual location was reported in a 42-year-old male who underwent wide local excision and the diagnosis was confirmed by histopathological and immunohistochemical investigations. It has been differentiated from the other differential diagnosis by immunohistochemistry. For any lesion with a polypoidal appearance, flesh-colored and firm in consistency, the diagnosis of superficial angiomyxoma needs to be ruled out. Since local recurrence is common, the patient needs to be kept under close follow-up.
